# Epidemiology of breast cancer: retrospective study in the Central African Republic

**DOI:** 10.1186/s12889-016-3863-6

**Published:** 2016-12-07

**Authors:** Augustin Balekouzou, Ping Yin, Christian Maucler Pamatika, Ghose Bishwajit, Sylvain Wilfrid Nambei, Marceline Djeintote, Barbara Esther Ouansaba, Chang Shu, Minghui Yin, Zhen Fu, Tingting Qing, Mingming Yan, Yuanli Chen, Hongyu Li, Zhongyu Xu, Boniface Koffi

**Affiliations:** 1 Department of Epidemiology and Biostatistics, School of Public Health, Tongji Medical College of Huazhong University of Sciences and Technology, Post Box Office 430030, Hangkong Road 13, Wuhan City, Hubei Province China; 2 Hospital Laboratory Friendship of Bangui, Avenue of independence, Bangui City, 4th disctrict Central African Republic; 3 School of Medicine and Health Management, Tongji Medical College of Huazhong University of Sciences and Technology, Post Box Office 430030, Wuhan, China; 4 National Laboratory of Clinical Biology and Public Health, Abdel Nasser Road, Post Box Office 1426, Bangui, Central African Republic; 5 Faculty of Health Sciences, University of Bangui, Avenue of the Martyrs, 18 Bangui city, 1st District Central African Republic

**Keywords:** Breast cancer, Women, Epidemiology, Histology, Bangui, Central African Republic

## Abstract

**Background:**

Breast cancer is recognised as a major public health problem in developing countries; however, there is very limited evidence about its epidemiology in the Central African Republic. The aim of this study was to investigate the epidemiological and histopathological characteristics of breast cancer in Bangui.

**Methods:**

This is a retrospective study based on the data collected from pathological anatomy records from 2003 to 2015 in Bangui. A questionnaire was designed to collect information and data was analysed using descriptive and inferential statistical methods.

**Results:**

The mean age was 45.83 (SD = 13.5) years. The age group of 45–54 years represented the majority of the study population (29.3%). Over 69.5% of the women were housewives with a moderate economic status (56.9%). Less than 14% of the study population had a level of academic degree and 85.6% lived in cities. The breast cancer prevalence was 15.27%. The age-standardized incidence and death by world population (ASW) were 11.19/100,000 and 9.97/100,000 respectively. 50–54 years were most affected. Left breast cancer is mainly common and the time between first symptoms and consultation is more than 48 months. Most (69%) of the samples analysed were lumpectomy. The most common morphology of breast cancer was invasive ductal carcinoma (64.9%). Scarff Bloom Richardson III was the main grade in both common pathological types, but their proportion showed no significant increase along with time (*χ*2 = 7.06, *p* = 0.54). Invasion of regional lymph node differed significantly among the pathological type of breast cancer (*χ*2 = 24.6, *p* = 0.02). Surgery and chemotherapy were appropriate treatment yet 84.5% of the cases died.

**Conclusion:**

The findings of this study showed that breast cancer is common and mostly affected women. Epidemiological trends are more or less common to those of developing countries with a predominance of invasive ductal carcinoma. However, most of the women studied live in an urban area and developed the disease in advanced stage. The establishment of an appropriate framework will effectively contribute to promoting the early detection and reducing the incidence of this disease in the population.

**Electronic supplementary material:**

The online version of this article (doi:10.1186/s12889-016-3863-6) contains supplementary material, which is available to authorized users.

## Background

Breast cancer (BC) is a common form of cancer among women globally. It’s the fifth leading cause of death with an average of 522,000 cases per year [1]. Currently, it is the most leading cause of cancer death with 198,000 deaths per annum which represents 15.4% of all deaths in developed regions after that of the lung cancer [2]. In developing countries, it is the first leading cause of death among women with 324,000 deaths which represented 14.3% of all deaths [2, 3]. Moreover, this rate varied from 6 to 20 per 100,000 in East Asia and West Africa as a whole [4].

In 2012, the number of new cases diagnosed in women was 1.7 million (25% of all cancers), with more cases observed in the developed regions (883,000 cases against 794,000 in developing countries) [4, 5]. Incidence rates almost quadrupled from one region to another throughout the world [4]. In 2012, the incidence rate in Africa and East Asia was 27 per 100,000, while in Europe it was 96 per 100,000 [4]. In 2011, the annual incidence rate in Sub-Saharan African (SSA) was 22 per 100,000 women [6]. The age distribution of the incidence of BC allows to objectifying a high incidence increased from 35 years, peaking at 60 [7].

The burden of the diseases caused by BC is important. However, BC etiology is far from being fully understood in developing countries such as Central African Republic (CAR). Large differences have been observed in the behaviour of the tumour, clinical manifestation, treatment response and prognosis [8].

According to WHO recent report estimates, the breast cancer-related mortality was the leading cause of death (24.5%) among Central African women in 2012 [9, 10].

The mortality rate of BC varies proportionally with age. It also depends on the stage of disease diagnosis, the speed of management, type and extent of the tumour, complacency and response to initial treatment. The main risk factors related to BC are hormonal factors related to pre-menopausal estrogenic impregnation, genetic predispositions, related factors behaviours and environmental factors [11].

In recent decades, CAR started to record a significant reduction in infectious diseases through the various national programs established. There was an epidemiological transition marked by demographic change, with an increase of life expectancy (50 years for men and 52 years for women), a transformation of the environment, and changes in lifestyle [9].

From this emerged, the new pathologies including cancer and other chronic non-communicable diseases which fall among the new priority public health needs [12]. Some studies have reported the clinical and pathological features of women BC in the CAR; however, there is no concrete evidence about the epidemiology of BC in the population [13–15]. Hence, the management of BC has become a major problem of public health which requires special attention from the ministry of health [16].

This study was conducted to address the lack of epidemiological evidence on BC, the absence of an operational plan for the fight against cancer and the lack of a support structure as well as national registry, with the main aim to understand the epidemiological characteristics of BC among women over 15 years old living in Bangui and to identify clinical histopathological trends in the last 12 years (2003–2015).

## Methods

### Study population

This was retrospective study based on the primary data collected through patient’s recorders from the pathological anatomy laboratory, surgical and gynecology services. The cases were confirmed by histological or cytological analysis during the study period. The following conditions were excluded: aged < 15 years old, not residing in CAR territory, and all those whose pathological diagnosis date was outside the study period. Research was approved by the Institutional Review Board of the School of Public Health, Tongji Medical College of Huazhong University of Science and Technology (IRB Approval File No.[2014] 09), and University of Bangui (No 2068/UB/FACSS/CSCVPER/16). Patient consent was not required due to the retrospective nature of this study.

### Data collection

A questionnaire was designed to collect information initially from the registrar of pathological anatomy laboratory; and from the medical records of patients in surgery and gynecology services who receive suspected cases. The information included: socio-demographic characteristics, such as age, occupation, economic status, education level, residence, ethnic group, marital status and parity; histo-pathological characteristics, such as location of the BC, delay between the first symptoms to consultation, nature of the sample, tumour type, tumour grade, treatment and evolution of the disease (Additional file 1).

### Classification criteria

Histology grade was based on Scarff-Bloom-Richardson grading system [17]. Economic status has been defined in terms of family income according to international poverty threshold. Income is poor if it is below 2 dollars a day, it is moderate between 2 and 4 dollars, good between 5 and 10 dollars and excellent if it exceeds 10 dollars [18]. The residence is called urban to those living in Bangui and rural for those living in the provinces (before diagnosis for cases). Ethnicity was grouped into seven major ethnic groups of the country.

### Statistical analysis

Data analysis was performed using the Statistical Package for Social Sciences (SPSS Inc., Chicago, IL, USA) version 20. Descriptive analysis was performed to characterize the demographic variables of the patients. Mean and standard deviation (SD) were described for the continuous variables with normal distribution and ranges for the continuous variables with skewed distribution. An independent *t*-test or nonparametric test was used to determine the difference between groups. Frequencies and proportions were used for categorical variables. The differences were determined by Chi-square test or a Fisher exact test. The analysis of variance method was used to compare the group differences. Trend test was used to detect the change trend of the related variables along with time. Statistical significance was set at the *P* value < 0.05.

## Results

### Socio-demographic characteristics of patients

In total, 174 cases of BC confirmed by pathological anatomy service were included in this study from 2003 to 2015. The socio-demographic characteristics of the patient were shown in Table 1. The age at diagnosis for the cases ranged from 16 to 90 years with a mean of 45.83 (SD = 13.5) years. The age group (45–54 years) represented the majority of the study population (29.3%). Over 69.5% of the sample was housewives with a moderate economic status (56.9%). Less than 14% of the study population had a level of academic degree and 85.6% lived in cities. The most represented ethnic groups were the Yakoma (21.8%), followed by Mandja (19.0%) and Banda (18.4%). The Mbororo ethnic was a minority with 1.1%. Single occupied 73.6% of the sample, however, a small proportion (17.9%) is nulliparous (Table 1).

**Table 1:** Socio-demographic characteristic of sample

Variables	Freq	Percent
Age group		
15–24	7	4.0
25–34	26	14.9
35–44	49	28.2
45–54	51	29.3
55–64	25	14.4
65–74	11	6.3
≥ 75	5	2.9
Mean ± Sdt (Mini, Maxi)	45.83 ± 13.55(16, 90)	
Occupation		
Housewife	121	69.5
Employer	53	30.5
Economic status		
Poor	24	13.8
Moderate	99	56.9
Good	48	27.6
Excellent	3	1.7
Education level		
None	45	25.9
Primary	57	32.8
Secondary	49	28.2
University	23	13.2
Residence		
Urban	149	85.6
Rural	25	14.4
Ethnic group		
Banda	32	18.4
Gbaya	22	12.6
Mandja	33	19.0
Sara	24	13.8
Yakoma	38	21.8
Mbororo	2	1.1
Ngbaka	23	13.2
Marital status		
Married	42	24.1
Single	128	73.6
Divorced/Widow	4	2.3
Parity		
Nulliparus	31	17.9
1–2	46	26.6
≥ 3	96	55.5

Frequency was calculated by using Cross tabulation analyze. Employee includes all sectors: public and private. Poor economic status (income < 2 dollars a day), moderate (income = 3 to 4 dollars a day), good (income = 5 to 10 dollars per day) and excellent (income > 15 dollars a day); Residence: Town (Bangui) and Rural (outside Bangui)

*Freq* Frequency, *Sdt* standard deviation

### Incidence and mortality rate of breast cancer

**Fig. 1:**
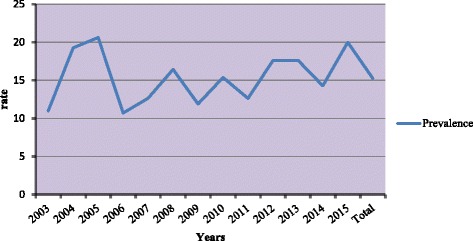
Prevalence of breast cancer among women per year from 2003 to 2015

**Fig. 2:**
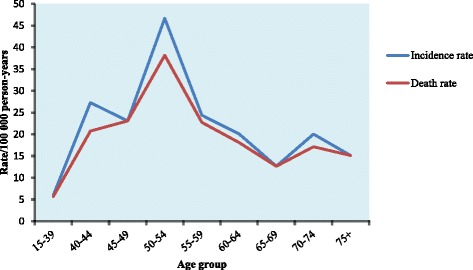
Age-adjusted Incidence and Death rates for Breast Cancer among Women over 15 years old in Central African Republic from 2003 to 2015

According to Fig. 1, the prevalence curve of BC is bimodal with two peaks at 2005 and 2015. The trend of incidence and mortality increased with age before 45 years and decreased after 55 years (Fig. 2). Three peaks were found when looked into age-specific incidences. The first peak appeared at the group of 40–44 years, and then the rate gradually decreased after 45. The second peak appeared at the group of 50–54 years and decreased after 55 years but dramatically increased again in the group 70–74 years and decreased over 75 years (Fig. 2).

Contrary to the incidence curve, the age-specific mortality had two peaks. The first peak appeared at the group of 50–54 years and the second was observed in the group of 70–74 years (Fig. 2). The annual average crude incidence was 7.49/100,000 women, while mortality was 6.71/100,000 women. After adjusted by standard population, the age-adjusted incidence rate for BC was 11.19 (95%, CI: 9.53–12.84) per 100,000 women. On the other hand, the age-adjusted death rate for BC was 9.97 (95%, CI: 8.86–12.17) per 100,000 women.

### Histo-pathological features

**Fig. 3:**
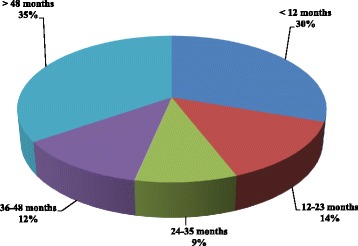
Delay between symptoms to consultation

**Fig. 4:**
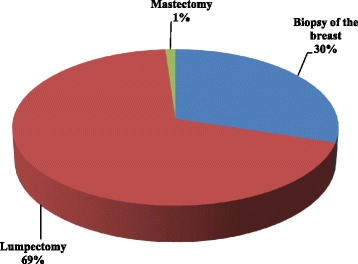
Nature of the sample

The left breast is mainly affected than the right breast (12% against 4%). The time between the first symptoms and the first consultation with a specialist was more than 48 months in 35% and less than 12 months in 30% of cases (Fig. 3). The majority of samples received and analysed by the pathological anatomy service consisted of 69% of lumpectomy followed by 30% breast biopsy (Fig. 4).

**Fig. 5:**
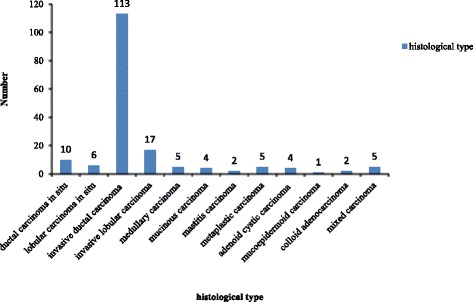
Distribution of tumours according to the histological type

The histological trend and grade of the common pathological types among the patients with BC are presented in Fig. 5 and Table 2. According to Fig. 5, the predominant tumour histological patterns were invasive ductal carcinoma 113 (64.9%), invasive lobular carcinoma 17 (9.8%) followed by ductal carcinoma in situ 10 (5.7%) and medullary carcinoma 5 (2.8%). On the other hand, the histological grade of Scarff Bloom Richardson (SBR) was available for 28 patients (16.1%). In our study, SBR III was the main grade in both common pathological types except for the lobular carcinoma in situ. In lobular carcinoma in situ and invasive lobular carcinoma, the proportion of SBR III was low, however, the proportion of SBR I was higher in lobular carcinoma than in situ. Unfortunately, the proportion of SBR III BC showed no significant increase along with time (*χ*2 = 7.06, *p* = 0.54). The proportion of invasion of regional lymph node differed significantly among the common pathological type of BC (*χ*2 = 24.6, *p* = 0.02) as in Table 2. According to Table 3, 166 (95.4%) of patients underwent surgery, and 159 (91.4%) were on chemotherapy. Radiology was performed in 53 (30.4%) women. However, the majority (84.5%) of the cases died instead, and only 21 (12.1%) remained alive. Note also that 6 (3.4%) were considered “lost to follow-up” in the investigation.

**Table 2:** Regional lymph node invasion of the common pathological type of breast cancer among all the patients

Type of Breast Cancer	N1	N2	N3	*χ*2	*P* value
	N (%)	N (%)	N (%)		
Ductal carcinoma in situ	107 (98.2)	2 (1.8)	0 (0.0)	24.6	*0.02*
Invasive ductal carcinoma	7 (87.5)	0 (0.0)	1 (12.5)		
Invasive lobular carcinoma	1 (50.0)	1 (50.0)	0 (0.0)		
Mucinous carcinoma	2 (100.0)	0 (0.0)	0 (0.0)		
Endocrine carcinoma of the breast	1 (100.0)	0 (0.0)	0 (0.0)		

*χ*2 and *p*-value were calculated by using Fisher’s exact chi square

The proportion of invasion of regional lymph node differed significantly among the common pathological type of BC (*χ*2=24.6, *p*=0.02)

*Freq* Frequency; *χ*2 chi square, *SD* Standard Deviation, *N* Lymph Node

**Table 3:** Distribution of the treatment and evolution of the disease

Treatment	Frequency	Percentage
Surgery		
Yes	166	95.4
No	8	4.6
Total	174	100
Chemotherapy		
Yes	159	91.4
No	15	8.6
Total	174	100
Radiology		
Yes	53	30.4
No	121	69.6
Total	174	100
Evolution		
Uncomplicated	21	12.1
Loss of view	6	3.4
Death	147	84.5
Total	174	100.0

## Discussion

The pathological diagnosis is very problematic in most African countries at the instance of the CAR. In CAR, there is only one pathological anatomy service located at Bangui the capital. This unique pathological anatomy service does not practice the essential immunohistochemistry to detect hormone receptors. However, for other molecular studies, this pathological anatomy service was forced to work with other European or American laboratories with the corollary examining tissue blocks stored in more or less good conditions. Unfortunately, we have no information on the results of these molecular analyses as the results have never been made public.

However, during the period of this study, 67.18% of women breast specimens received at pathological anatomy department were malignant. Most patients were in the age groups of 35 to 44 and 45 to 54 with 28.2% and 29.3% respectively. We can say that the predominant population found is consistent with some previous studies carried out worldwide [14, 15, 19, 20]. On the other hand, it should be noted that the majority of cases were diagnosed late; more than 48 months later. These findings confirm that BC in African women is characterised by a younger age at onset, advanced stage at diagnosis, and /or poor prognosis. For example, in our case, about one-third (*n* = 27; 18, 9%) of women with BC are in the group of younger age (15 to 34 years) and presented with the disease at an advanced stage. The reasons for the low sample size (174) and the late presentation of patients in hospitals include: lack of information and awareness about BC, lack of education, ignorance of the disease symptoms, cultural beliefs and stress disease, a sceptical attitude toward Western medicine and dependence on the traditional medicine. We cannot ignore the low socioeconomic level of the target population, the lack of access to quality care and a suitable deficit insufficient compared to the needs of technology and the lack of an effective national program for screening BC to promote weight reduction of this disease in the CAR population. This contrasts with that of the developed world where mammography screening and greater awareness of women had led to an early detection of the disease. The medico-social support accordingly ultimately results in increased survival rates of patients.

In addition, worldwide studies reveal that there is a higher incidence of BC in young women of African origin living in Western countries [21, 22]. This observation was confirmed by a study on the data collected from British women by Bown et al. who demonstrated that black British women were diagnosed with BC at a much younger age compared to their white counterparts [23]. In line with this, in Africa, a study by Ntekim et al. among young women living in Ibadan, Nigeria has reached the same conclusion [24].

Regarding the results of our study, the incidence rate reported for the period 2003 to 2015 is lower than that described in developed countries and some countries in Africa and Asia. According to the literature, the overall incidence of BC has increased in recent decades. It has reached more than 100 / 100,000 in developed countries, and more than 9% among American women have suffered from this disease in their lives [25]. In Asia and Africa, some countries have also experienced rapid growth in the incidence of BC. In China, for example, the reported incidence of BC for the period 2003–2007 was 68.6 / 100,000 in Shanghai and 57.6 / 100,000 in Beijing [26–28].

However, according to the WHO report in 2012, the incidence of BC is 31.5 / 100, 000 in Cameroon, 22.5 / 100,000 in Egypt, 24.2 / 100,000 in Ghana, 22.8 / 100,000 in Burkina Faso, 19.8 / 100,000 in Haiti and 15.9 / 100,000 in Guinea [4]. Among the population of Reunion Island, the crude incidence rate of the period of 2005 to 2010 for 20 to 49 aged women was 31.4 / 100,000. This lower incidence of BC in SSA among African women of a certain age can be explained by lower estrogen prolonged exposure (relatively late age of onset of menstruation, early pregnancy, the longest breastfeeding by the mother, multiparty, the rare practice of therapeutic hormone replacement during menopause). Thus, in a case-control study conducted in Nigeria by Huo et al. it has been demonstrated that multiparous women and those who practice breastfeeding for more than 12 months or more had a lower incidence of BC [29]. Some authors have reported similar results in South Africa [30]. But in a medical center in Nigeria in 1992, Ihekwala et al. have not found the same relationship [31]. According to the evolution of lifestyles, it is foreseeable that the epidemiology of BC in SSA would change in the coming decades. The change would be mainly due to a significant improvement in the level of education of women and their practice (use of oral contraception, first pregnancy before age 30, and abstention of certain substances that may cause BC, use of hormonal substitutions, obesity and physical inactivity).

On the other hand, the cumulative prevalence of BC in all cases of cancer diagnosed during the period 2003 to 2015 is 15.27%. This proportion is slightly below in countries such as Niger (19.28%), Madagascar (22.4%), Reunion Island (40%), Loire-Atlantique (24%), Isere (23.8%) among Caucasian - US (21%) and African American in the US (33.1%) [32, 33]. Moreover, the study indicates that a significant increase in BC incidence was observed among women 50 to 54 years. These results are different from those described by Marianne Dubard-Gault (2014) in Reunion Island and Bodmer et al. (2013) in Switzerland, where the incidence of BC has increased most in young women than those over 40 years old [34, 35]. Similarly in Europe during the period 1990 to 2009, incidence had increased among women aged under 35 years than those aged 35 to 39 years [35]. However, in the US, the incidence among young women under 40 years had increased by 1.47% per year [36].

Thus, some authors have reported that African women living in the urban areas lose the protective benefit of low estrogen exposure, age before their first pregnancy and menopause [37]. The same observation has been reported in a study by Ben Gobrane et al. and Ekortard et al. [38, 39]. These studies also explain the low proportion of patients living in rural area of the cohort. It could be also linked to the lack of qualified health workers at the peripheral level for refer them to the referral centers. Another reason was the distance from the capital Bangui where the only screening laboratory is available but inconsiderate of the low socioeconomic level of the CAR population. Sometimes people of the rural area which had the opportunity to go to the hospital are confronted by the weight of the traditional belief, which may justify their late submission to the service of modern medicine. Also, note that the significant increase of BC cases in the age group 45 to 54 confirm the priori literature indicating that BC is a disease of mature women.

The trend of mortality varies in proportion to the frequency in this study. However, the study noted a significant increase in the age group of 50 to 54. This rate is similar to that of Gabon (10.9 / 100,000), which is more than that of China (6.2 / 100,000) but lower than that of Cameroon, Egypt, Ghana and Haiti (25.7; 21.6; 18.5 and 14.2%’s per 100,000 respectively) [4]. According to the literature, the experiences of early detection of BC and starting treatment when the cancer is at a localized stage, significantly improve the prognosis of the disease and also contribute to a decline in mortality due to illness [40]. However, the high rates of BC mortality in this study could be attributable to delayed diagnosis or a lack of financial resources for health care, as published in the report of the World Bank in 2015. In fact, that report showed that more than 66% of the CAR population lived below the poverty line with 338.728 dollars per capita per year [41].

In our study, BC diagnosis is established after examination of parts of lumpectomy or breast biopsy. However, we noted that malignant tumours predominate in the left breast as reported in previous studies by Koffi et al. in CAR and Tre-Yavo et al. in Senegal respectively [14, 42]. Other studies, rather claim a predominance of cancer in the right breast [43]. The frequency of bilateral forms in our series is less than Mengué S. in Gabon or Khairy et al. in Saudi Arabia [43, 44]. Therefore, we do not have sufficient scientific evidence to justify the disparity of this BC location in CAR women.

In addition, the most common morphology of the BC (invasive ductal carcinoma) in our study was slightly below some studies by Darré et al. in Togo and Echimane et al. in Ivory Coast, which respectively reported 96 and 97% for cancers and 2.6 and 2.04% for sarcomas [45, 46]. While Engbang et al. in Cameroon and Der Muonir et al. in Ghana noted 74.3 and 91.6% of invasive ductal carcinomas followed by 4.3 and 3.1% of invasive lobular carcinoma [47, 48]. The same trends have been observed in previous studies by Koffi et al. in Bangui and have reported the prevalence of invasive ductal carcinoma followed by invasive lobular carcinoma [14].

Histoprognostic grade SBR studied in this series revealed a high proportion of grade III followed by grade II and grade I. In 2006, similar proportions were reported by Ohene et al. in Ghana [49]. These authors had found 53.7% of grade III tumors, 31.5% of grade II tumors and 14.8% of grade I tumor [49]. However, some studies have shown various proportions. A study by Essiben et al. found a trend in the following order of frequency: grades II, I and III to the Gynecological Obstetric and Pediatric Hospital in Yaoundé [50]. In 2004, Koffi et al. was in Bangui, noted that the grade II tumors were most significant with 58.4% of cases, while the tumor grade I and II were in the same proportion (20.8%) [14]. These findings are contrary to those reported in Libreville by Meye et al. that, the grade II held the last position after classes I and III [51].

Our study found that there is a statistically significant correlation between age and education level (*X* ^2^ = -0.155, *p* = 0.04). By cons, age and education level have no statistical relationship with the type of cancer (SBR). Similarly, between the women lived in rural area and the time at diagnosis. Even then, the chi-square of Pearson and Fisher test revealed no significant difference between age group and time for consultation of the doctor or type of cancer (SBR). This explains that early or late detection is independent of the level of education or age. But apparently, the level of knowledge about the symptoms of the disease and access to health care center is essential.

According to several studies, the conservative treatment of BC only affects some patients in SSA and only a few centers perform the sentinel lymph node technique [52, 53]. The most common surgical treatment is mastectomy with systematic lymphadenectomy. However, in our study, 95.2% of patients were treated with surgery and 91.4% with complementary chemotherapy. Our results were well above the studies by Gakwaya et al. were only 26% of patients received chemotherapy and Seredouma et al. of whom 66.3% had surgery [13, 54]. According to the socioeconomic context of the CAR, chemotherapy, although indicated for the treatment of BC, the prescription of anticancer drugs is often confronted by the availability of molecules on the one hand and the possibilities of financing by the patient. Despite the large number of patients receiving chemotherapy, only 21 (12.1%) of the patients were able to complete their treatment cycles. A cost of chemotherapy was estimated at $ 260, resulting in the cost of chemotherapy being varied from $ 1040 to $ 1560. This cost was well above the financial income of the patients according to the report of World Bank 2015 [41]. This justifies the difficulties of access to chemotherapy treatments whose drugs are not available in Bangui [55].

However, approximately 58.7% of patients in this series had no education or stopped at the elementary level. In addition, over 69% of the cohorts were housewives whose socio-economic status is low. Due to low levels of formal education, there is a lack of awareness of early warning signs of BC. Therefore, many patients died after the late diagnosis. Our study did not have sufficient information to calculate the survival rate of each positively diagnosed patient.

Some limitations of this study need to be addressed. First, it should be known that there are no population registers for cancer in CAR. Therefore, the incidence of BC per year could not be determined. The number of BC cases subjected to immune-histochemical analysis was not specified. The most concern is that there is no a broad national screening program for BC implemented in the country. From the points of view of the representativeness of this study data, the CAR has only pathological anatomy service for BC diagnosis and one cancerology unit for the treatment of all cases of the whole national territory. However, despite the small sample size, we believe that the results of this study can be extrapolated to the general target population of the CAR. Second, due to the retrospective nature of this study, a consecutive number of our data (14%) was excluded for lack of information as a result of the poor archives management. Third, a lack of information on the death date of patients which would allow us to calculate the survival rate after diagnosis. Nevertheless, we are convinced that the results and limitations of this study will contribute to the ongoing research in the field of BC in CAR and our comments would be replicated and verified on larger populations.

## Conclusion

According to the results of our study, BC disease is common and affected most of the women. Epidemiological trends are more or less common to those of developing countries, with a predominance of invasive ductal carcinoma. However, most of the women studied live in an urban area and developed the disease in advanced stage. It is obvious that the implementation of the National Cancer Registry could facilitate the study of the evolution of the tendency of cancer by age group in the future, to achieve an appropriate screening system and provide training to people at risk. This will help health officials monitor the disease in the community. Given that knowledge of BC trends and its common morphologies in the country can help to reduce its incidence rate in the population, via good planning.

## Additional file

Additional file 1: Questionnaire of study. (DOCX 354 kb)

## Abbreviations

BC: Breast Cancer
CAR: Central African Republic
SBR: Scarff Bloom Richardson
SPSS: Statistical Package for Social Sciences
SSA: Sub-Saharan African
WHO: World Health Organization
