# Supplementary material for: Epidemiology of breast cancer: retrospective study in the Central African Republic
Source: BMC Public Health. 2016 Dec 7;16:1230. doi: 10.1186/s12889-016-3863-6 (PMC5142143; doi:10.1186/s12889-016-3863-6)
Supplement: Additional file 1: — Questionnaire of study. (DOCX 354 kb) [file 12889_2016_3863_MOESM1_ESM.docx]

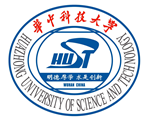


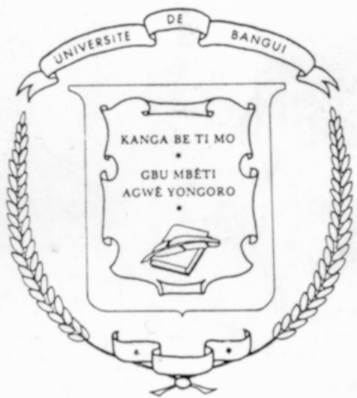


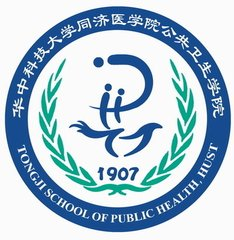


________________________________________________________________________________

**Questionnaire**

Doctoral Study "Public Health: Epidemiology and Biostatistics", Tongji Medical College, Huazhong University of Science and Technology, Wuhan, 40030, Hubei, China.

***Epidemiology of Breast cancer among women over 15 years living in Bangui, Central Africa Republic from 2003 to 2015***

N° inquiry form: ______________/ File Number: __________________/

1. **Personnel information**

A1. Patient Code: __________________________/ A2. Age (years): ____________________/

A3. Occupation: 1 2 77

1 = Housewife; 2 = Employer; 77 = Unknown

A4. Socioeconomic status of the family: 1 2 3 4 77

            1 = Poor; 2 = moderate; 3 = Good; 4 = Excellent; 77 = Indeterminate

A5. Ethnic group: 1 2 3 4 5 6 7 8 ……………77

1= Banda ; 2= Gbaya ; 3= Mandja ; 4= Sara ; 5= Ngbaka ; 6= Mbororo; 7= Yakoma ;8= Other (specify_________); 77 =Unknown

A6. Education level: 1 2 3 4 77

1 =None; 2 = Primary; 3= Secondary; 4= University; 77 = Unknown

A7. Residence: 1 2 77

1 = Urban; 2 = Rural; 77 = Unknown.

A8. Nationality: 1 2 77

1= Central African; 2 = Other (specify_________); 77= Unknown

A9. Marital status before diagnosed: 1 2 3 4 5 77

1 = Married; 2 = Single; 3 = Divorced; 4 = Widow; 5= other (specify________); 77= unknown

**B. Medical Records**

B1. Medical Treatment Centre: 1 2 3 4 5 6 77

1 = Reference Hospital; 2 = Regional Hospital; 3 = Reference center; 4 = Health Center
5 = Private clinic; 6 = Other (specify________); 77 = Unknown

B2. Delay for consultation: 1 2 3 4 5 77

**1=** < 12 months ; **2=** 12 – 23 months ; **3=** 24 – 35 months ; **4=** 36– 48 months ; **5=** > 48 months ; 77= Unknown

**C. Cytological Anatomy Data**

C.1. Examination requested by anapath: 1 2 3 4 5 77

**1** = Patient herself; **2** = Doctor; **3** = Surgeon; **4** = Gynecologist; **5**= Other (specify____); **77**= Unknown

C2. FNA (Cytoponction ): 1 2 3 4 4 77

**1** = Cancer; **2** = benign tumor; **3** = Normal; **4**= Other (specify_______); **77**= Unknown.

C3. Diagnostic date: _________________________/

C4. Nature of the sample sent for examination anapath: 1 2 3 4 5

1 = Biopsy of the breast; 2 = Lumpectomy; 3 = Mastectomy; 4 = Lymph node dissection; 5= 3 + 4

C5. Histological type : 1 2 3 4 5 6 7 8 9 10

11 12 13 15 16 17 18

1 = ductal carcinoma in situ; 2 = lobular carcinoma in situ; 3 = invasive ductal carcinoma ; 4 = invasive lobular carcinoma; 5 = tubular carcinoma; 6 = medullary carcinoma; 7 = mucinous carcinoma; 8 = cribriform infiltrating carcinoma; 9 = endocrine carcinoma of the breast; 10 = metaplastic carcinoma; 11 = apocrine carcinoma; 12 = adenoid cystic carcinoma; 13 = mucoepidermoid carcinoma; 14 = secreting carcinoma; 15 = micropappillaire infiltrating carcinoma; 16 = malignant phyllodes tumor; 17 = scirrhous carcinoma; 18 = colloidal adenocarcinoma

C6. Lymph node histology: 1 2 3 1 = Not done; 2 = flooding; 3 = No invasion

C7. SBR Rating: 1 2 3 (1= Grade I; 2= Grade II; 3= Grade III)

C8. Cytological type : 1 2 3 4 5 6 7 8 9 10

11 12 13 15 16 17 18

1 = ductal carcinoma in situ; 2 = lobular carcinoma in situ; 3 = invasive ductal carcinoma ; 4 = invasive lobular carcinoma; 5 = tubular carcinoma; 6 = medullary carcinoma; 7 = mucinous carcinoma; 8 = cribriform infiltrating carcinoma; 9 = endocrine carcinoma of the breast; 10 = metaplastic carcinoma; 11 = apocrine carcinoma; 12 = adenoid cystic carcinoma; 13 = mucoepidermoid carcinoma; 14 = secreting carcinoma; 15 = micropappillaire infiltrating carcinoma; 16 = malignant phyllodes tumor; 17 = scirrhous carcinoma; 18 = adenocarcinoma

**D. Treatment**

D1.1. Surgery: 1 2 77 (**0**= Yes; **1**= No; **77**= Unknown)

D1.2. Chemotherapy: 1 2 77 (**0**= Yes; **1**= No; **77**= Unknown)

D 1.3. Radiotherapy: 1 2 77 (**0**= Yes; **1**= No; **77**= Unknown)

D 1.4. Hormone: 1 2 77 (**0**= Yes; **1**= No; **77**= Unknown)

**E. Evolution**

E1. Evolution: 1 2 3 4 77

**1** = Healing; **2** = Complications; **3** = Loss of view; **4** = Death; **77**= Unknown

*If death to continue thereafter. If No death stop*

E2: Death date: ……./……../………. ( year /month/date)
